# Elevated Expression of lncRNA *MEG3* Induces Endothelial Dysfunction on HUVECs of IVF Born Offspring via Epigenetic Regulation

**DOI:** 10.3389/fcvm.2021.717729

**Published:** 2022-01-03

**Authors:** Ying Jiang, Hong Zhu, Hong Chen, Yi-Chen Yu, Ye-Tao Xu, Fang Liu, Sai-Nan He, Matthew Sagnelli, Yi-Min Zhu, Qiong Luo

**Affiliations:** ^1^Department of Obstetrics, School of Medicine, Women's Hospital, Zhejiang University, Hangzhou, China; ^2^Institute of Reproduction and Development, Obstetrics and Gynecology Hospital, Fudan University, Shanghai, China; ^3^Shanghai Ji Ai Genetics and IVF Institute, Institute of Reproduction and Development, Obstetrics and Gynecology Hospital, Fudan University, Shanghai, China; ^4^Regional Community Health Service Center of Minzhi Sub-district, Shenzhen, China; ^5^Department of General Surgery, School of Medicine, Sir Run Run Shaw Hospital, Zhejiang University, Hangzhou, China; ^6^Department of Obstetrics and Gynecology, The First Affiliated Hospital of Nanjing Medical University, Nanjing, China; ^7^Department of Cardiology, Beijing Chaoyang Hospital, Capital Medical University, Beijing, China; ^8^University of Connecticut School of Medicine, Farmington, CT, United States

**Keywords:** long non-coding RNA, endothelial dysfunction, IVF, epigenetic, fetal-origin adult diseases

## Abstract

Cardiovascular dysfunction in children born after *in vitro* fertilization (IVF) has been of great concern, the potential molecular mechanisms for such long-term outcomes are still unknown. Here, we found that systolic blood pressure was a little higher in IVF born offspring at 2 years old compared to those born after being naturally conceived. Besides, the expression level of maternally expressed gene 3 (*MEG3*) was higher in human umbilical vein endothelial cells (HUVECs) from IVF offspring than that in spontaneously born offspring. Pearson correlation test showed that *MEG3* relative expression is significantly related to the children's blood pressure (Coefficient = 0.429, *P* = 0.0262). Furthermore, we found decreased expression of endothelial nitric oxide synthase (*eNOS*) and vascular endothelial growth factor *(VEGF)* along with elevated expression of endothelial-1(*ET1*) in HUVECs from IVF offspring, accompanied by lower secretion of nitrite, *VEGF*, and higher secretion of *ET1* in the umbilical cord serum of IVF offspring. Correlation analysis showed MEG3 expression highly correlated with ET1 and Nitrate concentration. With pyrosequencing technology, we found that elevated expression of *MEG3* was the result of hypomethylation of the *MEG3* promoter. Therefore, our results provide a potential mechanism addressing the high-risk of hypertension in IVF offspring via MEG3 epigenetic regulation.

## Introduction

Since the first *in vitro* fertilization (IVF) pregnancy was reported in 1978 (1), an estimated seven million pregnancies have been achieved worldwide by IVF. Recently, an increasing number of studies have shown that IVF conceived fetuses are exposed to a high-estradiol environment *in utero*, which is closely correlated with increased risk of low birth weight (LBW) and small-for-gestational-age (SGA) (2, 3). Disturbed intrauterine environments have been proven to be associated with rapid weight gain in early childhood, increased risk of high blood pressure in late childhood, a higher number of skin folds, and elevated fasting serum glucose level concentrations (4, 5). In our laboratory, we also previously reported that, at the age of 3–13 years old, the blood pressure of IVF-conceived Chinese children was higher than that of spontaneously conceived born children (3). While recent studies have focused on the epidemiological consequences of IVF conceived offspring, few have explored the potential molecular mechanisms for such outcomes.

The “Fetal programming hypothesis” proposed by Barker and Fall suggests that cardiovascular and related disorders derive from fetal adaptions to adverse *in utero* environments, which could permanently alter offspring's postnatal metabolism and physiology (6). For example, maternal undernutrition induced intrauterine growth retardation, which causes a significant decrease in the number of nephrons within 1 year of birth, could be an underlying mechanism in the early development of hypertension (7, 8). However, the question as to how an intrauterine high-estradiol environment increases the risk of hypertension in IVF offspring later in life remains contentious (4, 9, 10).

Epigenetic modifications, such as DNA methylation, histone modifications, and non-coding RNAs are involved in mediating how early life impacts later health. Long non-coding RNAs (lncRNAs) are non-coding transcripts that are longer than 200 nucleotides in length. So far, abundant studies have demonstrated that dysfunction of lncRNAs is associated with the pathogenesis and progression of a broad range of diseases including cardiovascular disease (11), examples of this include cyclin-dependent kinase inhibitor 2B (*CDKN2B*) antisense RNA 1 (*ANRIL*), which have been implicated in atherosclerosis (12), and metastasis associated lung adenocarcinoma transcript 1(*MALAT1*), which has been shown to stimulate vascular growth *in vivo* and drive the proliferation of migratory endothelial cells *in vitro* (13). *MEG3* is a long non-coding RNA located at chromosome 14q32.3 in humans. *MEG3* is expressed in many normal tissues, and lost in several human tumors and tumor cell lines (14). *MEG3* is also expressed in arterial endothelial cells (13), and *MEG3* knock-out enhances the expression of *VEGF* signaling pathway genes in the brain (15). Notably, *MEG3* is the only significantly increased lncRNA in senescent HUVEC, which suggests that *MEG3* may mediate endothelial dysfunction in aging (16). Hypoxic conditions result in a significantly increased *MEG3* expression, accompanied by endothelial cell proliferation, migration and angiogenesis, cell death, and growth arrest (17). Furthermore, the abnormal methylation status of *MEG3* contributes to vascular defects, which induce abnormal placentation (18).

In this study, we investigated the role of lncRNA *MEG3* in the umbilical cord blood vessels of IVF born offspring, which might offer a potential mechanism for adult chronic cardiovascular diseases of fetal origin.

## Methods

### Study Population

We reviewed the records of 421 singletons born by natural conception and 482 singletons born by *in vitro* fertilization-embryo transfer (IVF-ET) in our hospital between 2013 and 2016. We evaluated the fetal growth measurement and ratio of umbilical cord systolic peak velocity over end diastolic velocity (S/D). Furthermore, we recruited 21 singletons born by fresh IVF-ET and 22 singletons who were naturally conceived (NC) from January 1, 2016, to January 1, 2017, for evaluation of the function of human umbilical endothelial cells. The baselines of parental characteristics were collected in the third trimester. These included maternal blood pressure, heart rate, the serum levels of fasting blood glucose, triglycerides, total cholesterol, high-density lipoprotein cholesterol, low-density lipoprotein cholesterol, homocysteine, and serum estradiol concentration.

Children born with cardiovascular diseases, congenital anomalies, premature delivery, who were small for gestational age were excluded. We also excluded children whose mothers had gestational complications such as gestational diabetes, pre-eclampsia, and others.

### Tissue Samples

Umbilical vessels and cord blood were collected from twenty-one IVF and twenty-two NC singleton pregnancies immediately after cesarean delivery in the Women's Hospital. The detailed characteristics of these samples are listed in Table 1. Mothers with previous cardiovascular diseases or other gestational complications were also excluded. Mothers of IVF babies had a normal ovarian function and experienced controlled ovarian hyper-stimulation with gonadotropins followed by the standard luteal long gonadotropin-releasing hormone agonist down-regulation protocol for the first IVF cycle. Embryo transfer was performed after 2–3 days of egg retrieval. All babies included met the following criteria: maternal age between 25 and 35 years old; full-term delivery; singleton pregnancy; child's birth weight was between 2,500 and 4,000 g; no indication of pregnancy complication; no birth defects; and no cardiovascular diseases. We examined the E2 levels in cord blood from newborns with the E2 kit (H-Estradiol E2, Abcam, ab108640).

**Table 1:** The characteristic of the maternal characteristics at the admission to hospital, children at birth, and 2 years-old blood pressure follow up.

**Characteristics**	**NC (*N* = 22)**	**IVF (*N* = 21)**	***P*-value**
Maternal age, yr	33.82 ± 3.05	32.71 ± 3.35	0.69
BMI before pregnancy, kg/m^2^	20.87 ± 2.58	22.03 ± 1.56	0.09
BMI before delivery, kg/m^2^	26.49 ± 2.11	28.39 ± 2.02	<0.01
Gestational age, wk	38.00 ± 0.61	38.00 ± 1.60	>0.99
Systolic BP, mmHg	117.1 ± 10.2	122.5 ± 10.7	0.10
Diastolic BP, mmHg	73.23 ± 7.70	75.9 ± 10.67	0.35
Heart rate, bpm	89.27 ± 12.19	90.86 ± 10.35	0.65
Random blood sugar, mmol/L	5.2 ± 0.98	5.34 ± 1.19	0.64
Triglycerides, mmol/L	3.83 ± 3.05	4.96 ± 3.02	0.23
Total cholesterol, mmol/L	5.90 ± 1.45	6.23 ± 1.60	0.23
HDL cholesterol, mmol/L	1.58 ± 0.42	1.74 ± 0.36	0.18
LDL cholesterol, mmol/L	2.47 ± 0.66	3.06 ± 0.73	<0.01
Homocysteic acid,mmol/L	6.94 ± 1.50	7.66 ± 1.51	0.12
Male birth, *n*, %	54.5 ± 10.8%	47.6 ± 11.1%	0.66
Birth weight, g	3,424 ± 411.5	3,204 ± 675.0	0.20
Pregnancy complications or cardiovascular disease risk in the mother	No	No	
Bi-pariental diameters,	9.23 ± 0.37	9.19 ± 0.45	0.27
Femur diameters	7.0 ± 0.45	6.85 ± 0.51	0.18
Blood estradiol concentration (pg/ml)	3,246 ± 209.1	3,773 ± 197.6	P < 0.05
Children systolic blood pressure around 2 years old	80.7 ± 4.85	84.1 ± 5.23	P < 0.05
Children diastolic blood pressure around 2 years old	49.28 ± 5.00	50.12 ± 5.67	0.15

*Data are presented as Mean ± SD, NC, naturally conceived; IVF, in vitro fertilization. Pregnancy complications include diabetes mellitus, arterial hypertension, pre-eclampsia, and dyslipidemia*.

### Isolation and Culture of Primary Human Umbilical Vein Endothelial Cells

The protocols for HUVEC isolation and culture were performed and modified according to Crampton et al. (19). Briefly, the fresh vein was filled with a solution containing 1 mg/ml collagenase. The cord was incubated in pre-warmed phosphate-buffered saline at 37°C for 30 min, and cells were cultured in 5% fetal bovine serum combined with endothelial cell medium (ECM, ScienCell, cat. #1001). Over 90% of HUVECs in one plate under the microscope were considered to indicate the successful isolation of the HUVECs used between passages 3–5.

### Small Interfering RNA-Mediated MEG3 Knock-Down

SiRNA oligonucleotides were purchased from Thermo Fisher Scientific. The sequences of siRNAs targeting *MEG3* are as follows (5′-3′): sense, GCUCAUACUUUGACUCUAUTT; anti-sense, AUAGAGUCAAAGUAUGAGCTT. The sequences of negative control (NC) are as follows (5′-3′): sense, UUCUCCGAACGUGUCACGUdTdT; anti-sense, ACGUGACACGUUCGGAGAAdTdT. The siRNAs against *MEG3* were reverse-transfected into cells at a dose of 10 nm in 6-wells-plates using the Lipofectamine 3000 reagent (ThermoFisher, Catalog. L3000008) for 48 h.

### Quantitative Realtime PCR Analysis

Total RNA was extracted from the tissue sample and primary human umbilical vessel cells using the TRIzol Reagents (Invitrogen Life Technologies, Carlsbad, CA, USA). cDNA was synthesized using PrimerScript RT Reagent Kit (Takara, RR037A, Japan) in a 20 μl reaction containing 0.5–1 ug of total RNA. Real-time quantitative polymerase chain reaction (RT-qPCR) was performed using ABI Prism 7900HT (Applied Biosystem, Foster City, CA). Glyceraldehyde-3-Phosphate Dehydrogenase (GAPDH) was the internal control. The full list of primer sequences is shown in Supplementary Table 1.

### Western Blot

The protein was extracted from HUVECs tissues with lysis buffer, which was separated using 10% SDS-PAGE. Western Blots was performed using polyvinylidene fluoride membrane and the antibodies for DNA Methyltransferase 3 Alpha (DNMT3A) (Cell Signaling, 32578, used at a dilution of 1:1000), DNA Methyltransferase 3 Alpha (DNA Methyltransferase 3 Beta) (Cell Signaling, 57868, used at a dilution of 1:1000), beta-ACTIN (Abcam, ab8226, used at a dilution of 1:5000). Protein bands were visualized by the enhanced chemiluminescence system (Pierce, Rockford, IL).

### ELISA Assay

Umbilical cord blood and cell supernatants were collected after cesarean delivery. ELISA kits were used for the determination of NO (Nitric Oxide Assay, ab65328, Abcam, UK), VEGF (H-VEGF, DVE00, R&D, USA), and ET-1 (H-Endothelin-1, QET00B, R&D, USA) levels. The procedures were performed according to the manufacturer's protocols. For the cell-line culture, three replicates of data were used for statistical analyses.

### DNA Isolation and Bisulfite Conversion

Total genomic DNA was isolated from umbilical vessel tissues using Genomic DNA Purification Kit (Invitrogen, cat. K0512, USA). Bisulfite was converted using the EpiTect bisulfite kit (Qiagen, Valencia, CA) according to the manufacturer's instructions to deaminate cytosine to uracil; 5-methyl-cytosine was protected from deamination. PCRs were performed in an ABI 9700 PCR System (Applied Biosystems, USA) using an annealing temperature of 56°C.

### DNA Methylation Analysis by Pyrosequencing

The bisulfite converted DNA was amplified using Hotstart Plus DNA polymerase (Qiagen). PCR products were immobilized on streptavidin-sepharose beads (GE Healthcare), washed, denatured, and released into annealing buffer containing sequencing primer, which is described in Supplementary Table 2. Pyrosequencing was carried out on a PyroMark Q96 instrument (Qiagen) according to the manufacturer's instructions. Percent methylation was calculated using the Pyro Q CpG software (Qiagen).

### Statistical Analysis

Data were analyzed using SPSS 18.0 and were presented as mean ± SD. Statistical analysis including unpaired two-tailed Student's *t*-test was performed as described in the figure legends or Excel legends. Correlations of *MEG3* relative expression with the children's blood pressure at 2 years old were performed using the Pearson correlation coefficient. *P* < 0.05, *P* < 0.01, or *P* < 0.001 was considered statistically significant.

## Results

### Perinatal Characteristics and Children' Blood Pressure at 2 Years-Old

The baseline and perinatal characteristics of the study population are shown in Table 1. Children's systolic blood pressure (SBE) at 2 years old, born by fresh IVF-ET was slightly higher compared with those who were naturally conceived (84.1 ± 5.23 vs. 80.7 ± 4.85, *P* < 0.05), while there were no differences in the aspect of diastolic blood pressure and birth weight. Besides, by reviewing the baseline and perinatal characteristics of 903 babies (421 NC babies and 482 IVF babies) who were born in the past 4 years (2013–2016) in our hospital (Table 2), we found that there were no significant differences in S/D ratio, which are related to umbilical cord vessel function. Additionally, bi-parietal, femur diameter, and birth weight were smaller in IVF born babies (9.16 ± 0.44 vs. 9.26 ± 0.41, *P* < 0.01; 6.88 ± 0.44 vs. 7.02 ± 0.41, *P* < 0.0001; 3,250 ± 478.1 vs. 3,183 ± 643.3, *P* < 0.05), which might be induced by earlier delivery (37.48 ± 2.41 vs. 38.44 ± 1.84, *P* < 0.0001; Table 2).

**Table 2:** The characteristic of children at birth, fetal development index (36–38 weeks ultrasound), and the maternal characteristics at conception.

**Characteristics**	**NC (*N* = 421)**	**IVF (*N* = 482)**	***P*-value**
Maternal age, yr	30.49 ± 3.69	30.85 ± 3.95	0.34
BMI before delivery, kg/m^2^	22.56 ± 2.25	25.32 ± 3.69	0.23
Gestational age, wk	38.44 ± 1.84	37.48 ± 2.41	<0.0001
Systolic BP, mmHg	119.7 ± 11.90	120.9 ± 14.7	0.16
Diastolic BP, mmHg	74.49 ± 7.38	75.57 ± 9.44	0.06
Heart rate, bpm	86.29 ± 8.40	87.4 ± 9.00	0.06
random blood sugar, mmol/L	4.761 ± 1.23	4.94 ± 1.32	<0.05(0.04)
Triglycerides, mmol/L	3.06 ± 1.84	3.73 ± 1.98	<0.0001
Total cholesterol, mmol/L	6.19 ± 1.23	6.28 ± 1.31	0.27
HDL cholesterol, mmol/L	1.81 ± 0.40	1.74 ± 0.44	<0.05(0.01)
LDL cholesterol, mmol/L	2.95 ± 0.82	2.82 ± 0.88	0.06
Male birth, *n*, %	52.0 ± 2.4%	49.6 ± 2.2%	0.49
Birth weight, g	3,250 ± 478.1	3,183 ± 643.3	<0.05
Bi-parietal diameter, cm	9.26 ± 0.41	9.16 ± 0.44	<0.01(0.003)
Femur diameter, cm	7.02 ± 0.41	6.88 ± 0.44	<0.0001
S/D ratio	2.174 ± 0.41	2.191 ± 0.44	0.50

*Data are presented as Mean ± SD, NC, naturally conceived; IVF, in vitro fertilization. Women with Pregnancy complications including diabetes mellitus, arterial hypertension, and dyslipidemia were excluded*.

### Up-Regulated Expression of *MEG3* in IVF HUVEC

**Figure 1:**
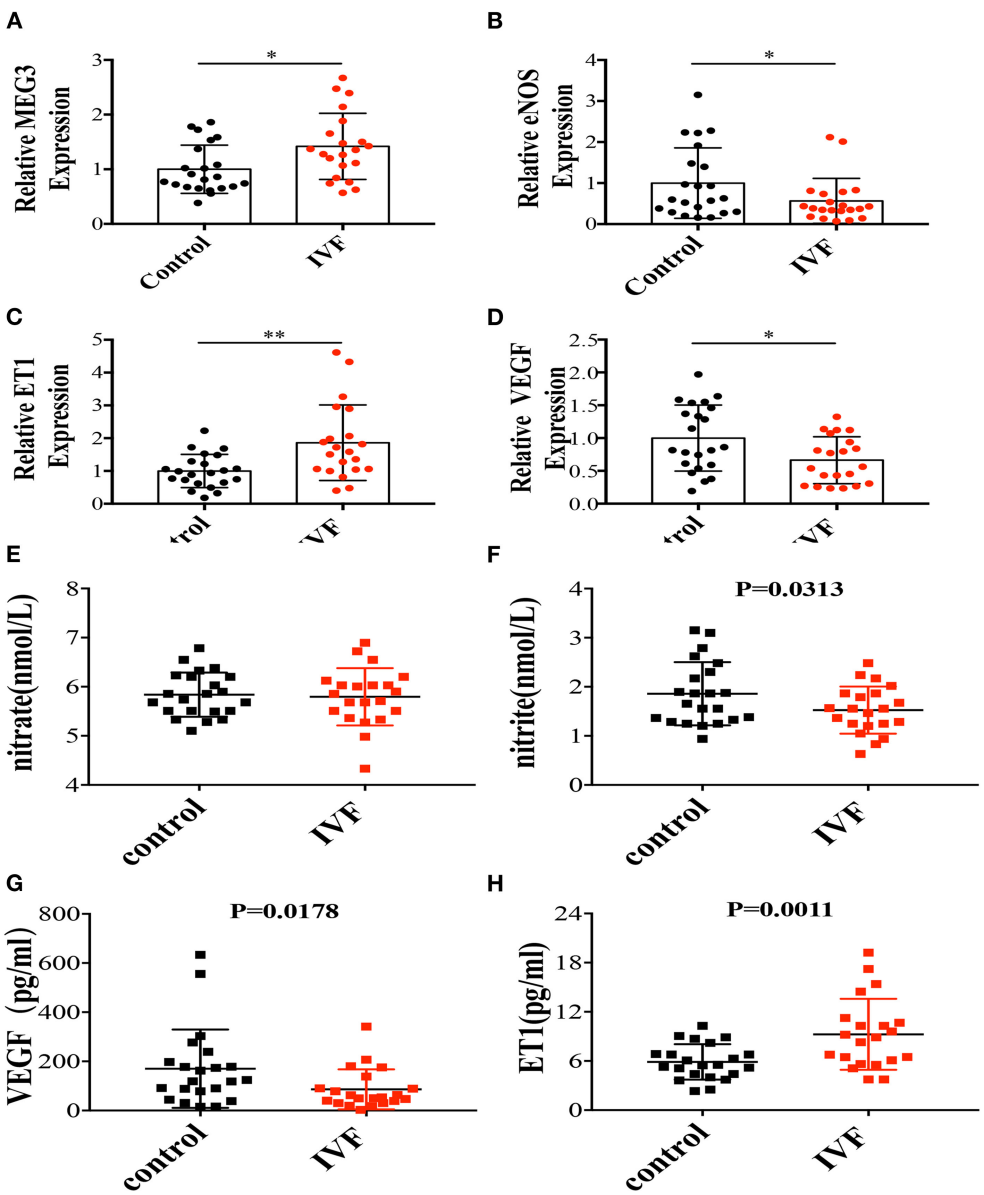
The mRNA expressions of MEG3 and endothelium-derived genes and serum secretions related with endothelial function. **(A–D)** RNA levels determined by RT-qPCR. Data were analyzed with the Equation 2^−ΔΔCT^,where ΔΔCT = ΔCT (treatment group) –ΔCT (control group), and ΔCT = CT (sample)–CT (internal control). The values were normalized to GAPDH mRNA levels. **(E–H)** ELISA was performed to detect the secretion of nitrate **(E)**, nitrite **(F)**, VEGF **(G)**, and ET1 **(H)**. Control has 22 samples and IVF group has 21 samples. In all panels, data are presented as mean ± SD, *P < 0.05, **P < 0.01. Significance was determined by Student *t*-test.

To identify the mRNA expression level of *MEG3* in HUVEC, we found that the expression level of *MEG3* was significantly higher in IVF born HUVECs (Figure 1A) by qPCR. This suggests that the intrauterine environment of IVF induced abnormal *MEG3* expression.

### Endothelium-Derived Factors Altered in HUVEC of IVF Offspring

We previously found that offspring born after IVF have elevated blood pressure between the ages of 3–13 years old (3). This study observed similar results in offspring around 2 years of age (Table 1). Therefore, we focused on the endothelium-derived factors, such as endothelial nitric oxide synthase (*eNOS*), which is a classical vasodilation factor, endothelin-1(*ET1*), one of the critical vasoconstriction factors, and vascular endothelial growth factor (*VEGF*), which is related with endothelial cell proliferation, angiogenesis, and vascular permeability. qPCR showed that *eNOS* and *VEGF* expression were significantly decreased in the IVF offspring group (Figures 1B,D), while *ET1* expression was significantly increased (Figure 1C). Since NO has a very short half-life of several seconds, using ELISA, we tested the levels of the first and second oxidation products of NO, nitrite and nitrate, respectively. The results showed that nitrite concentration was significantly lower in the umbilical cord serum of IVF offspring (Figure 1F), while there was no difference in nitrate concentration (Figure 1E). Furthermore, *ET1* was significantly higher in IVF offspring (Figure 1H) while *VEGF* secretion was decreased (Figure 1G), which is coincident with the mRNA level. These results suggested that the intra-uterine environment of IVF might lead to endothelial dysfunction associated with vascular activity since HUVECs share a common embryological origin with other fetal vessels.

### Knockdown of *MEG3* in HUVEC Decreases *ET1* Expression and Increases Secretion of Nitrite and VEGF *in vitro*

**Figure 2:**
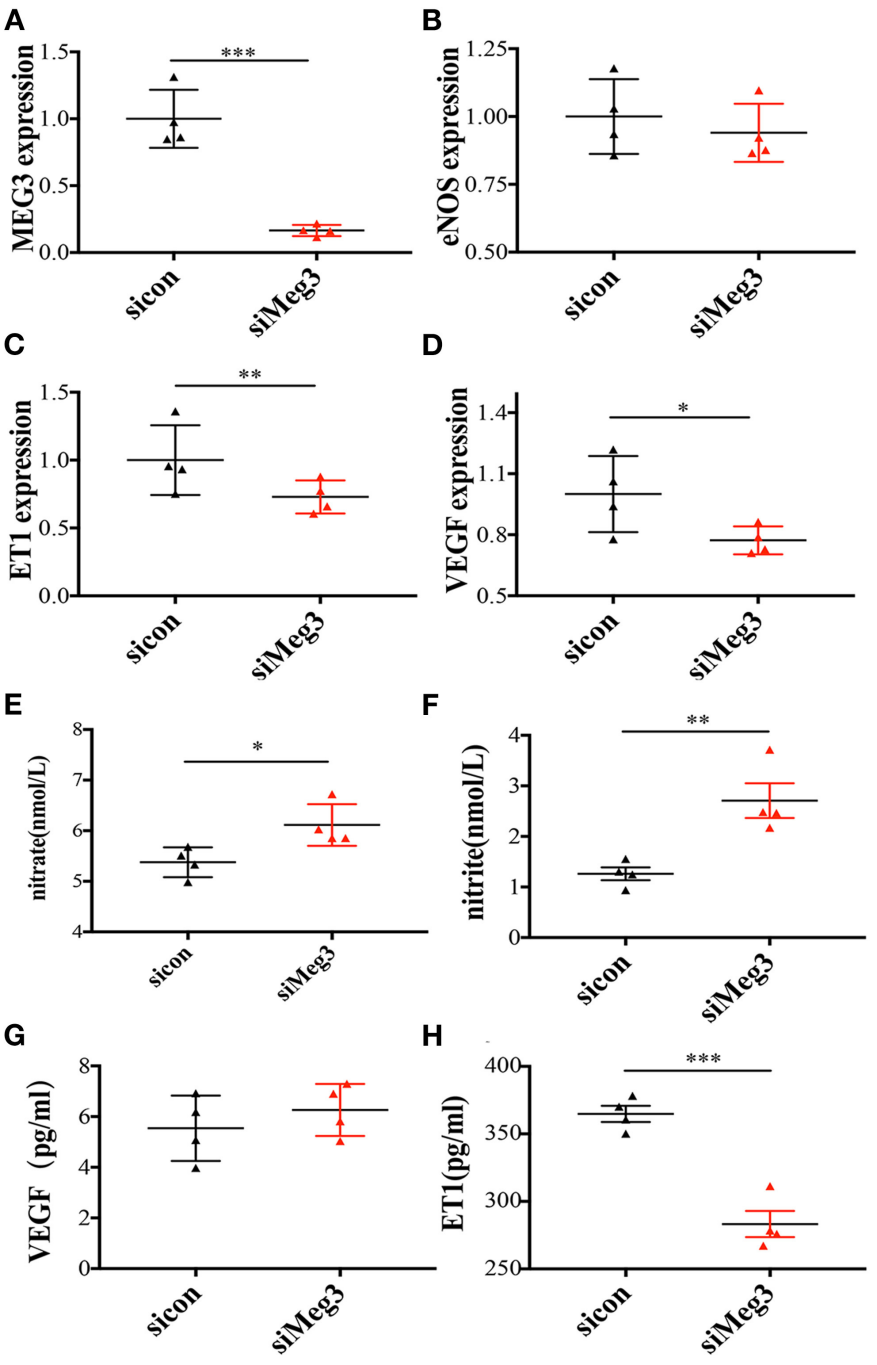
The mRNA expression of endothelium-derived genes in MEG3 knockdown treatment *in-vitro* primary human umbilical endothelial cells and serum secretions of these target proteins in supernatant liquids. **(A–D)** RNA levels determined by RT-qPCR. Data were analyzed with the Equation 2^−ΔΔCT^, where ΔΔCT = ΔCT (treatment group) –ΔCT (control group), and ΔCT = ΔCT (sample)–ΔCT (internal control). The values were normalized to GAPDH mRNA levels. **(E–H)** ELISA was performed to detect the secretion of nitrate **(E)**, nitrite **(F)**, VEGF **(G)**, and ET1 **(H)**. Both control and IVF group have 4 samples and tried for triple times. In all panels, data are presented as mean ± SD, *P < 0.05, **P < 0.01, ***P < 0.001. Significance was determined by Student *t*-test.

Michalik et al. reported that *MEG3* was highly rich in the nuclear fraction, and profoundly increased by hypoxia (13). Given this data, we further explored the function of *MEG3* in endothelial cells by silencing *MEG3* expression with one specific siRNA, which could reduce endogenously expressed *MEG3* by 85% (Figure 2A). Through silencing *MEG3*, we discovered that *MEG3* reduction significantly suppressed *ET1* mRNA levels and *VEGF* mRNA levels (Figures 2C,D), but did not arouse any significant change in *eNOS* expression (Figure 2B). Through ELISA, we demonstrated that nitrite and nitrite secretion was significantly increased with *MEG3* suppression (Figures 2E,F), while *ET1* secretion was decreased (Figure 2H). We also observed no significant difference in *VEGF* secretion with *MEG3* suppression (Figure 2G), which was consistent with the mRNA synthesis, and might be related to the diverse stimulation mechanisms of VEGF production (20).

### High Expression Level of *MEG3* in Human Umbilical Vein Endothelial Cells Was Controlled by DNA Methylation

**Figure 3:**
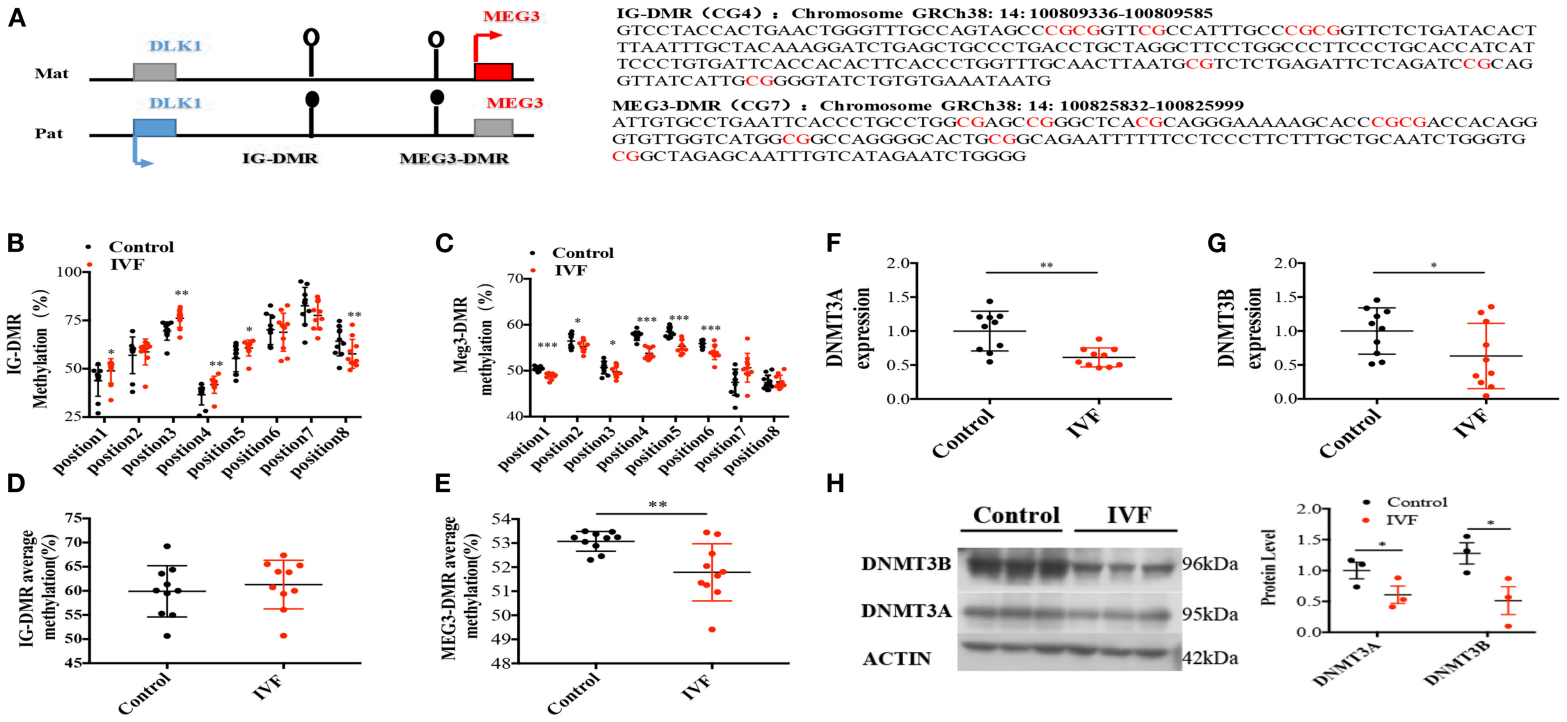
Methylation analysis of IG-DMR/MEG3-DMR by pyrosequencing. **(A)** Schematic representation of human imprinted locus, showing the relative position of the DLK1 and MEG3 genes and indicating the location of the two DMRs known to contribute to MEG3 imprinting. Exons are known as blue (DLK1 gene) and red (MEG3 gene) rectangles with arrows for transcription start sites. Under the genetic map listed specific gene sequence of the two DMRs. **(B)** Methylation status of individual DNA strands of IG-DMR containing 8 CpG sites and the average methylation ratio in each CpG site; **(C)** Methylation status of individual DNA strands of MEG3-DMR containing 8 CpG sites and the average methylation ratio in each CpG site. **(D,E)** The average methylation status of IG-DMR and MEG3-DMR, ten patients were included in each CpG site. **(F,G)** Dnmt3A, Dnmt3B RNA levels determined by RT-qPCR. **(H)** Dnmt3A, Dnmt3B protein levels determined by Western-Blot. Data were analyzed with the Equation 2^−ΔΔCT^, where ΔΔCT = ΔCT (treatment group) –ΔCT (control group), and ΔCT = ΔCT (sample)–ΔCT (internal control). The values were normalized to GAPDH mRNA levels. For western blot, the values were normalized to β-ACTIN protein levels. For pyrosequencing and RT-qPCR, there were 10 samples both for control and IVF group, for Western-blot, there were 3 samples both for control and IVF group. In all panels, data are presented as mean ± SD, *P < 0.05, **P < 0.01, ***P < 0.001. Significance was determined by Student *t*-test.

As a maternally imprinted gene, *MEG3* expression is regulated by two critical different methylation regions (DMR): the intergenic region between *DLK1* and *MEG3* (IG-DMR), and MEG3 DMR (CG7) (21) (Figure 3A). Pyrosequencing showed no difference in the average methylation status in IG-DMR between IVF offspring and spontaneously born offspring (Figure 3D), while 5 sites were in hypermethylation status from the aspect of single CpG status in the IG-DMR (Figure 3B). However, MEG3-DMR, located in the −287 to −120 region of the human *MEG3* promoter (which has eight differentially methylated CpGs), showed significantly reduced methylation status in IVF HUVECs, which consisted of 6 sites of hypomethylation (Figures 3C,E).

Three DNMTs determine the methylation status of DNA methylation in humans. DNMT3a and DNMT3b are responsible for *de novo* methylation, while DNMT1 is conservatively expressed and required for maintenance of methylation (22). We performed qPCR and Western-blot to detect the expression of DNMTs, and the results indicated that there was a significant reduction of DNMT3A and DNMT3B in IVF HUVECs compared with spontaneous-born HUVECs (Figures 3F–H) both in mRNA level and protein levels, which was consistent with the DNA methylation status of *MEG3* DMR.

### Abnormal *MEG3* Expression Might Be the Inducer of Endothelial Dysfunction

**Figure 4:**
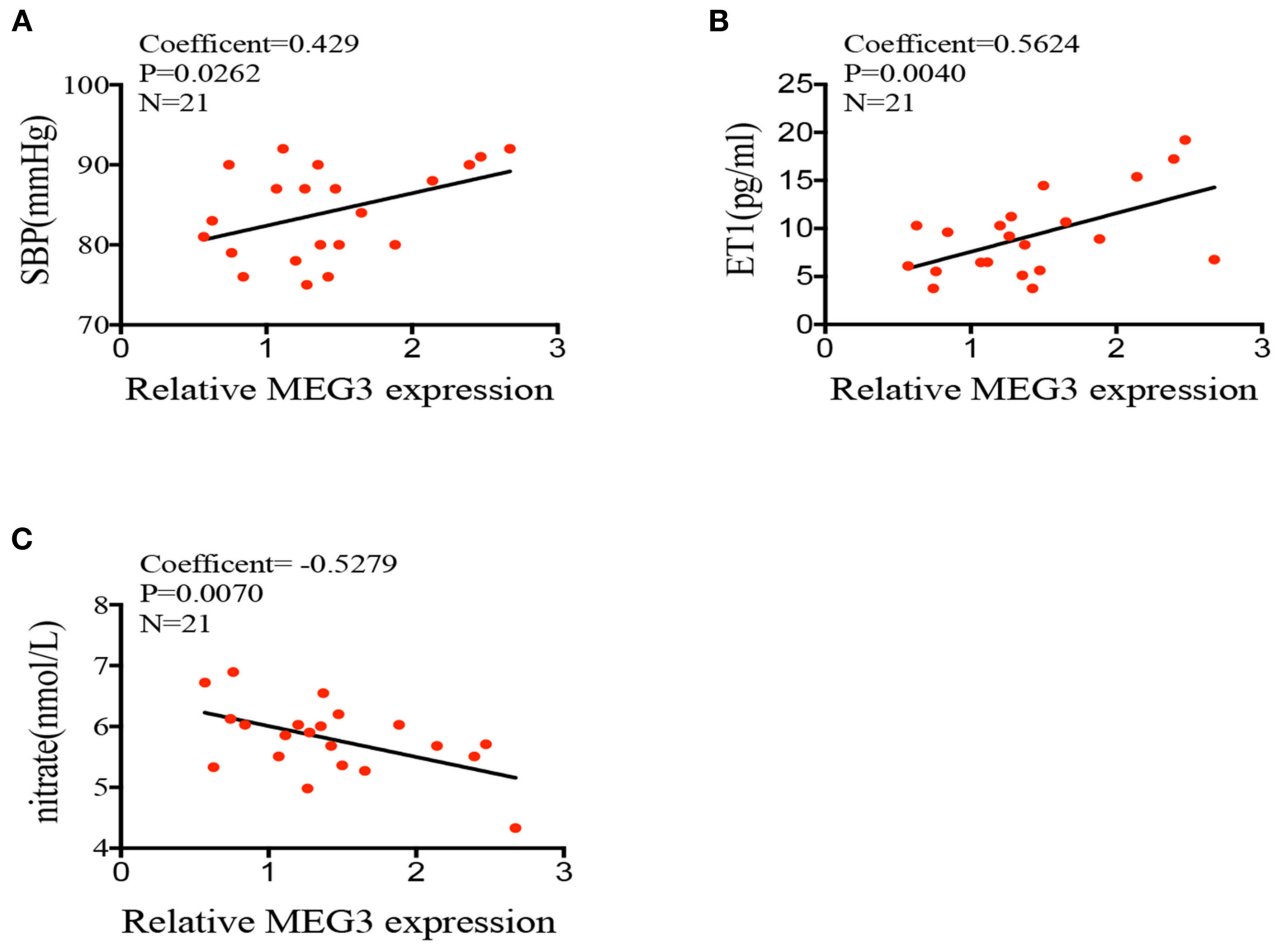
Correlations between MEG3 expression and endothelial-derived factors. **(A–C)** correlation between MEG3 relative expression and children's blood pressure, serum nitrate, ET1 concentration. Significance was determined by Pearson correlation coefficient.

To support the idea that elevated expression of long non-coding RNA *MEG3* leads to abnormal endothelial function, we compared the *MEG3* expression on HUVECs derived from IVF born offspring and children's blood pressure at 2 years old. Pearson correlation test showed that *MEG3* relative expression was significantly related to the children's blood pressure (Coefficient = 0.429, *P* = 0.0262; Figure 4A). Furthermore, we also analyzed the correlation of *MEG3* expression and endothelial-derived factors in IVF born umbilical cords. In line with the observed higher expression of *MEG3* in HUVECs of IVF born offspring, *MEG3* expression was also significantly negatively related with nitrate concentration (Coefficient = −0.5279, *P* = 0.0070; Figure 4C), while positively connected with *ET1* concentration (Coefficient = 0.5624, *P* = 0.0040; Figure 4B).

### Effects of High Estradiol Concentration on Primary Human Umbilical Vein Cells

**Figure 5:**
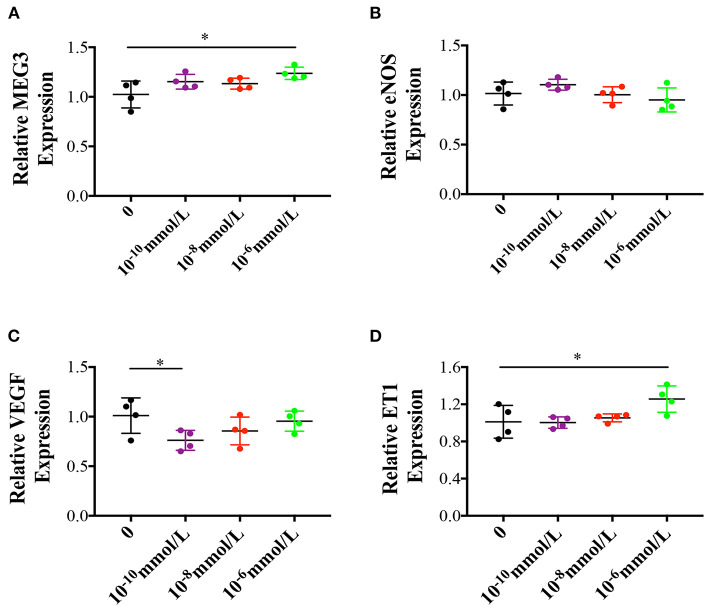
Effects of different estradiol concentration on primary human umbilical vein endothelial cells. **(A–D)** Represented MEG3, eNOS, VEGF, and ET1 RNA levels which were determined by RT-qPCR. The values were normalized to GAPDH mRNA levels. For different estradiol treatment group, there were 4 samples and tried for triple times. In all panels, data are presented as mean ± SD, *P < 0.05, **P < 0.01. Significance was determined by Student *t*-test.

In order to understand the cause of the up-regulation of MEG3, we cultured primary HUVECs in three different estradiol concentrations (10^−10^, 10^−8^, 10^−6^ mmol/L). We found that as estradiol concentration went up, MEG3 and ET1 expression were significantly increased (Figures 5A,D) while eNOS and VEGF expression were decreased (Figures 5B,C), which were in accordance with the *in vivo* results.

## Discussion

This study is the first to demonstrate that long coding RNA *MEG3* suppresses vasodilation while promoting vasoconstriction in HUVECs derived from IVF born offspring. Our molecular findings were consistent with follow-up data showing that IVF offspring have a higher incidence of hypertension. Meanwhile, these alterations may originate from the regulation of MEG3-DMR at the epigenetic level, leading to higher expression of *MEG3*. This study may provide a novel mechanism and potential theory for high risk of hypertension in IVF offspring.

The endothelium is one of the largest organ systems by surface area. Normal endothelial function is critical in keeping the symbiotic balance between vasoconstrictive (namely like *ET1*) and vasodilatory (namely via NO) stimuli, thus a disturbance in normal endothelial function is a predictor of future adverse cardiovascular events (23). Since endothelial cells from the cord may reflect characteristics of the offspring's vascular system (24). In our study, we sought to investigate the function of HUVECs derived from IVF born babies. As we know, the endothelial-dependent response to vasodilate is fundamentally regulated by a release of nitric oxide (NO) synthesized from the amino-acid L-arginine by endothelial nitric oxidase synthase (*eNOS*) (25). In addition to NO as one critical factor in endothelial function, enhanced activities and levels of *ET1* also were related to endothelial dysfunction by stimulating nicotinamide adenine dinucleotide phosphate (NADPH) oxidase-derived ROS production, which inhibits NO-mediated endothelial relaxation and mediating ET_A_ receptors to blunt NO relaxant responses (26). *VEGF* plays a central role in endothelial function, including stimulating endothelial proliferation, migration, and nitric oxide release. Thus, *VEGF* might exhibit the same tendency with NO (27). In our study, we found that the IVF group has lower *eNOS* and *VEGF* expression, lower secretion of nitrate, nitrite, *VEGF* concentration, and higher *ET1* expression and production, all of which might lead to endothelial dysfunction in IVF born babies. Although certain concentration and time course exposures of estradiol may improve NO product and thus enhance vasodilation (28), as observed in our study, it may be related to extremely elevated concentrations of estradiol *in vitro* and long-term estradiol exposure *in vivo* for IVF born babies, which may result in endothelial wall damage.

The recent identification of a novel group of mediators known as long-coding RNAs (lncRNAs) has provided a large quantity of new biology to explore for cardiovascular risk reduction. Several lncRNAs take part in acute myocardial infarction (eg, Novlnc6), heart failure (eg, Myosin Heavy Chain Associated RNA Transcript, *Mhrt*), control hypertrophy and apoptosis of cardiomyocytes (29), and the regulation of vascular growth and function (e.g., *MALAT1)* (13). *MEG3* is an imprinted gene belonging to the imprinted *DLK1-MEG3* locus at chromosome 14q32.3 in humans. The gene expression in this locus is tightly controlled by at least two differentially methylation regions (DMRs): the intergenic DMR (IG-DMR) and the MEG3-DMR. Numerous studies have implicated the involvement of *MEG3* in a myriad of biological processes, notably as a tumor suppressor (14, 30). *MEG3* is also involved in many cardiovascular functions, including angiogenesis through *VEGFA* and *VEGF1R* expression (31, 32) and smooth muscle cell proliferation through the p53 pathway. Knocking-down MEG3 with a nano-polymer wrapped MEG3 short hairpin RNA (shRNA) plasmid conjugated with OX26 antibody (MPO) enhanced endothelial cells migration, tube formation *in vitro* and reduced the volume of cerebral infarction, capillary density, cerebral cortex micro-vessel *in vivo* by increasing the angiogenesis associated genes VEGFA and VEGFR (33). In an *in vivo* mice model of hindlimb ischemia, MEG3 inhibition increased blood flow recovery (16). Besides, MEG3 acts as a miRNA sponge in vascular ECs by negatively regulating miR-9, a key player in angiogenesis and proliferation (34). In this study, we show that HUVECs from IVF born offspring have high expression levels of *MEG3*. Besides, *MEG3* expression was significantly correlated with endothelial-derived factors, including nitrate and ET1, and long-term children's blood pressure at 2 years old. Taken together, these results suggest that *MEG3* may be involved in the etiology of cardiovascular diseases of offspring born in an intrauterine high-estradiol environment.

## Conclusion

Altogether these data show that IVF neonates have an abnormal endothelium response in human umbilical vein endothelial cells, decreased *eNOS* expression and synthesis of NO in endothelial cells, and increased *ET1* expression and secretion in umbilical cord serum, which may be the result of elevated expression of *MEG3*. These data suggest that the abnormal expression of *MEG3* may contribute to the development of cardiovascular disease in IVF born offspring later in life.

## Data Availability Statement

The datasets presented in this study can be found in online repositories. The names of the repository/repositories and accession number(s) can be found at: 10.5061/dryad.hhmgqnkhc.

## Ethics Statement

The study protocol (ethical review serial number: 20180046) was approved by the Research and Ethics Committee of the Women's Hospital, School of Medicine, Zhejiang University, China. All participants signed informed consents. We design our study in accordance with the Declaration of Helsinki.

## Author Contributions

YJ, HZ, and HC contributed to collection, analysis, and interpretation of data as well as manuscript preparation. YC-Y contributed to data collection and analysis. SN-H, YT-X, and FL contributed to interpretation of data. MS contributed to edit the language. QL and YM-Z contributed to study design and data interpretation and the manuscript preparation. QL is the guarantor of this work and, as such, has full access to all the data in the study and takes responsibility for the integrity of the data and the accuracy of the data analysis.

## Funding

This work was supported by the Key Subjects Group of Reproductive Medicine, School of Medicine, Zhejiang University. It was funded by the National Key R&D Program of China (2018YFC1002702), the National Nature Science Foundation of China (Grant Nos. 81571447 and 81501339), Construction of Medical Core Subjects and Innovation Platform in Zhejiang Province (Grant No. 2018RC005), the Medical and Health Technology Program (General Project) in Zhejiang Province (Grant Nos. 2014KYA245 and 2016KYB150), the Fundamental Research Funds for the Central Universities (2018FZA7011), and the Natural Scientific Foundation of Zhejiang Province (LY13H040004).

## Conflict of Interest

The authors declare that the research was conducted in the absence of any commercial or financial relationships that could be construed as a potential conflict of interest.

## Publisher's Note

All claims expressed in this article are solely those of the authors and do not necessarily represent those of their affiliated organizations, or those of the publisher, the editors and the reviewers. Any product that may be evaluated in this article, or claim that may be made by its manufacturer, is not guaranteed or endorsed by the publisher.
