# Supplementary material for: Elevated Expression of lncRNA MEG3 Induces Endothelial Dysfunction on HUVECs of IVF Born Offspring via Epigenetic Regulation
Source: Front Cardiovasc Med. 2022 Jan 3;8:717729. doi: 10.3389/fcvm.2021.717729 (PMC8761900; doi:10.3389/fcvm.2021.717729)
Supplement: Supplementary file 1 [file Table_1.DOCX]

**Supplementary Table 1. Nucleotide sequences of primers (human) used for real-time quantitative PCR (SYBR Green)**

| Target RNA | Primers (5’ to 3’ direction) | Product Size |
| --- | --- | --- |
| GAPDH | GGGAAACTGTGGCGTGAT | 308bp |
|  | AAGGTGGAGGAGTGGGTGT |  |
| Meg3 | ACTCGCTCTACTCCGTGGAA | 215bp |
|  | CACATTCGAGGTCCCTTCCC |  |
| VEGF | CGAAGAGAAGAGACACATTG | 197bp |
|  | GGATGGAGGAAGGTCAAC |  |
| DNMT3A | TCAGGTTAAGCCAGCAATT | 200bp |
|  | ATCATACACGCTTGGAAGG |  |
| DNMT3B | CTTGGAGCAGCCTAACAC | 171bp |
|  | CATTAACCTGAGTATTGGAGAG |  |
| eNOS | TGGAGTGGTTTGCAGCCCT | 346bp |
|  | TGCTCATTCTCCAGGTGCTTCA |  |
| ET1 | GCCTGTCTGAAGCCATAG | 236bp |
|  | GAGAGGTCCATTGTCATCC |  |

**Supplementary Table 2 Nucleotide sequences of primers (human) used for pryosequencing PCR**

| Methylation Analysis | Forward Primer | Reverse Primer | AT |
| --- | --- | --- | --- |
| CG4 F/R | TTTTATTATTGAATTGGGTTTGTTAGT | ACAATTCCTACTACAAAATTTCAACA | 57 |
| CG7 F/R | TTGTGTTTGAATTTATTTTGTTT | CCCCAAATTCTATAACAAATTACT | 57 |
