# Supplementary material for: Elevated Expression of lncRNA MEG3 Induces Endothelial Dysfunction on HUVECs of IVF Born Offspring via Epigenetic Regulation
Source: Front Cardiovasc Med. 2022 Jan 3;8:717729. doi: 10.3389/fcvm.2021.717729 (PMC8761900; doi:10.3389/fcvm.2021.717729)
Supplement: Supplementary file 2 [file Data_Sheet_1.docx]

**Women’s Hospital, School of Medicine, Zhejiang University**

**Ethical Review and Approval Documents**

Ethical review serial number：20180046

| Protocol Name | The long coding RNA Meg3 takes part in the effect of intrauterine hyper-estradiol environment on the offspring’s cardiovascular system | | |
| --- | --- | --- | --- |
| Items of application | Clinical scientific research | Major of application | Obstetrics |
| Project research unit | Women’s Hospital, School of Medicine, Zhejiang University | | |
| The lead unit | Women’s Hospital, School of Medicine, Zhejiang University | | |
| agency responsibility | Ying Jiang | Technical Post | Physician |
| Principal investigator | Qiong Luo | Technical Post | Attending Physician |
| major investigators | Qiong Luo | Technical Post | Attending Physician |
| Materials of review | Research Programmer  Informed consent | Approach of review | Review |
| Contact information of Ethics committee | Address: women’s hospital, school of medicine, Zhejiang university, No.1, Xueshi Road, Shangcheng District, Hangzhou, China, 310006  Tel: +86 571 87061501 | | |
| Signed by attended members of Ethics committee | Details as attached list | | |
| Review comment | | | |
| This study performed in accordance with the ethical standards. The board of ethics committee agreed to the study work as planned.  Review opinion and suggestion: No □ Yes□  Does the process of this study accept the review of Ethics Committee? Yes□ No□  The frequency of the review as of the date of approval: 3 months□ 6 months□ 1 year □  The Ethics Committee shall have the right to change the review frequency according to the actual progress of this study.  Signed by chairman of The Ethics Committee: Date: | | | |
| Attention:  1. The approval document shall valid for 3 years, once over the period of validity, please send the application again to continue the study.  2. This document will be filing for the record in every central agencies and its ethics committee. Please inform the legal status of Institutional Review Board, if the committees have different opinion about the project's feasibility in the institution.  3.The project should executed obey the approved protocol by The Ethics Committee. This study was performed in accordance with SFDA-GCP and the ethical standards laid down in the Declaration of Helsinki.  4. Please report to the legal status of Institutional Review Board  Termination of clinical research if the clinical research was suspended or stopped in advance.  5. Please report the ethics committee promptly if some serious adverse events and unexpected events which affect the risk benefit ratio occurs.  6.Any alteration of the approved documents such as Clinical Protocols and informed consent, as well as principal investigator should be informed to the ethics committee. The plan could be carried out only after the review and approval.  7. The situation infringe experiment scheme should be reported to the ethics committee.  8. According to the ethics committee's opinion on the frequency of the continues review，please apply in 1 month before the deadline of the continues review.  9. Please submit the concluding report to the ethics committee for review when the clinical research completed. | | | |
